# A novel score to evaluate nonlinear association between insulin resistance and new-onset diabetes mellitus: A cohort analysis

**DOI:** 10.1097/MD.0000000000050092

**Published:** 2026-08-07

**Authors:** Baojia Zhang

**Affiliations:** a Zhangye People’s Hospital Affiliated with Hexi College, Zhangye, Gansu Province, P.R. China.

**Keywords:** diabetes mellitus, metabolic score for insulin resistance, predictive analyses, prospective cohort study

## Abstract

The rising global prevalence of diabetes mellitus (DM) highlights the need for early risk assessment to delay disease onset. This study aims to evaluate the association between metabolic score for insulin resistance (Mets-IR) and the risk of new-onset DM. Data were obtained from the China Health and Retirement Longitudinal Study database. Participants aged <50 years or with missing data were excluded. Using 2011 as the baseline, the study endpoint was defined as the occurrence of new-onset DM during follow-up until 2018. The association between Mets-IR and new-onset DM risk was assessed using logistic regression models. Subgroup and sensitivity analyses were performed to evaluate robustness. Nonlinear associations were explored through threshold effect analysis and smoothed curve fitting. A total of 5257 people were included. Mets-IR was 1 unit per liter, and the risk of new-onset diabetes increased by 1%. The highest quartile risk was the lowest 5.67 times, showing a dose–response relationship. The threshold is 49.3, and the following risks rise sharply by 11% per unit. Subgroups showed that those under 65 years old, married, rural, nonsmokers, and those without liver and kidney diseases were more associated. The nonlinear association remains after comprehensive adjustment for confounders, suggesting that Mets-IR may provide a valuable additional tool for DM risk stratification and early intervention.

## 1. Introduction

Diabetes mellitus (DM) is a major global chronic disease, with type 2 DM constituting the majority of cases.^[1,2]^ Current projections indicate the global population with DM will exceed 1.3 billion by 2050; in China, the incidence of DM remains high, driven by ongoing urbanization and population aging.^[3]^ The progression of DM leads to severe complications, such as renal insufficiency, retinopathy, and amputation,^[4–6]^ which impose great physical and psychological burdens on patients and increase public healthcare expenditure.^[7]^ Early prediction of DM and targeted interventions for high-risk groups can reduce or delay the incidence of DM. The identification of safe, effective, and affordable screening tools can provide useful information for clinical practice and public health policy-making, potentially reducing healthcare costs.^[8]^

Insulin resistance is a core pathophysiological feature of prediabetes and a key precursor to overt DM.^[9–14]^ Although the hyperinsulinemic-euglycemic clamp is the gold standard for measuring insulin sensitivity, its cost, complexity, and invasiveness limit its use in routine practice and large-scale screening. Therefore, it is urgent to find an alternative that is convenient, inexpensive, and safe. The Metabolic Score for Insulin Resistance (Mets-IR) is a recently developed index for assessing insulin sensitivity. Recent studies have shown associations between Mets-IR and various diseases such as cardiovascular disease, hypertension, stroke, and depression.^[15–25]^ However, evidence regarding its association with new-onset DM, particularly from large-scale longitudinal studies, remains limited.

Therefore, this study aims to investigate the relationship between Mets-IR and new-onset DM through a large-sample study, the China Health and Retirement Longitudinal Study (CHARLS), which will provide a valuable scientific basis for the prevention of DM.

## 2. Methods

### 2.1. Study design and participants

This study utilized data from CHARLS, a nationally representative longitudinal study of participants from 150 counties or districts and 450 villages in 28 provinces in China over a 10-year follow-up period starting in 2011. CHARLS focuses on the health and socioeconomic status of China’s middle-aged and elderly population. The current analysis used 2011 as the baseline, with follow-up continuing through 2018.

**Figure 1.**
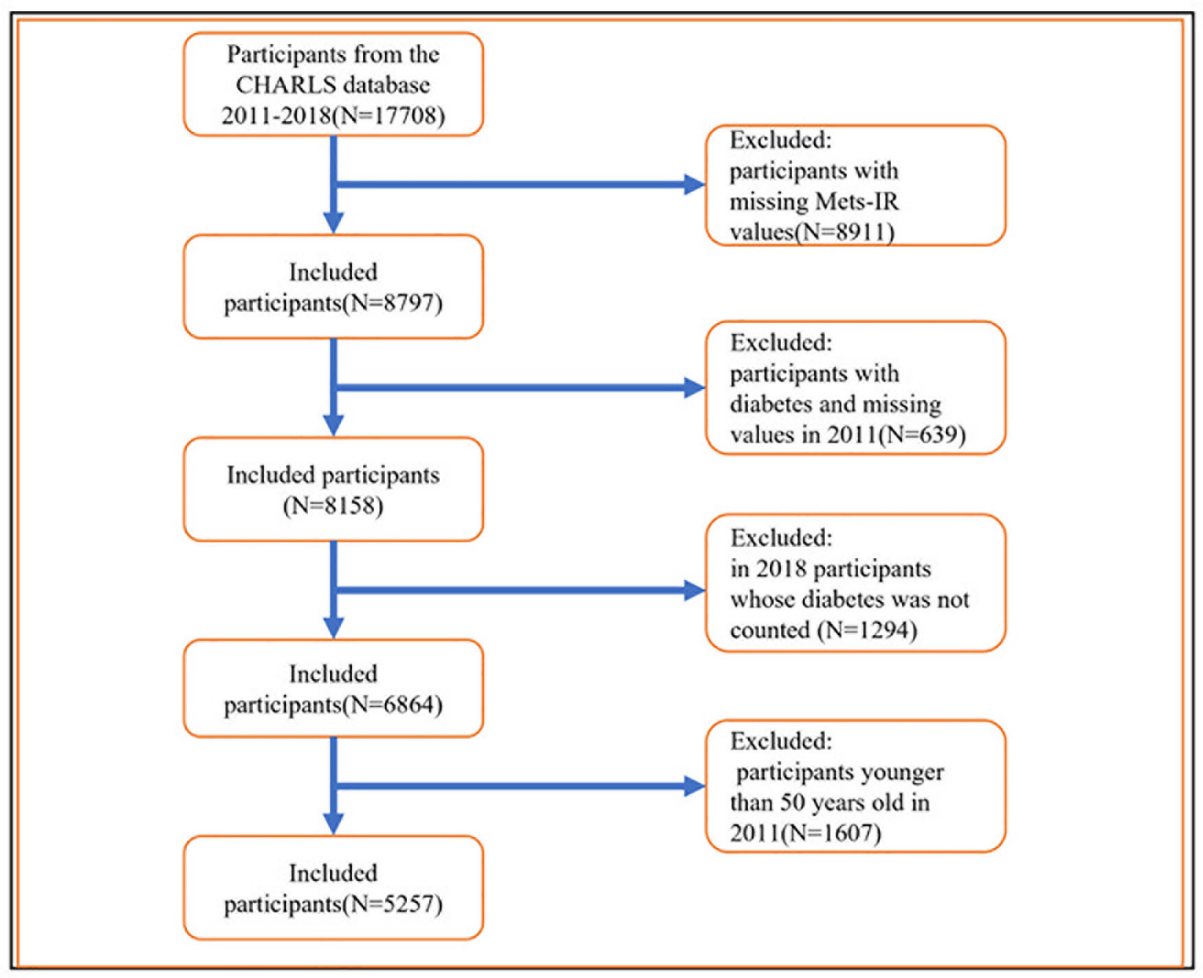
Flowchart of participant screening. CHARLS = China Health and Retirement Longitudinal Study, Mets-IR = metabolic score for insulin resistance.

Participants were included if they were ≥50 years of age and completed the initial medical examination. Individuals were excluded from the study if they had missing data (e.g., blood biomarkers for Mets-IR). Respondents with missing information during follow-up were also excluded. Figure 1 details the participant selection flowchart, resulting in a final analytical sample of 5257 individuals.

### 2.2. Data collection and covariates

#### 2.2.1. Covariates

Clinical and laboratory data included participants’ body mass index (BMI), age, marital status, education, gender, drinking and smoking status, and place of residence. Based on the CHARLS database, this study categorized smoking status as “never-smokers,” “former smokers,” or “current smokers.” For analysis, “former” and “current” smokers were combined into “smokers.” Alcohol consumption was defined as drinking ≥12 times or more per year, with responses recorded as “yes” or “no. This classification method has been used in previous large-scale epidemiological studies and can effectively distinguish exposure status.^[26]^

#### 2.2.2. Metabolic score for insulin resistance

Mets-IR was calculated using fasting blood biomarkers and BMI according to the following formula: Mets-IR = Ln (2 × fasting plasma glucose  + triglycerides) × BMI)/Ln (high-density lipoprotein cholesterol).

#### 2.2.3. Diabetes mellitus

Based on the self-reported medical history from the CHARLS questionnaire, individuals who answered “yes” to the item “Has a doctor ever told you that you have diabetes or elevated blood glucose (including impaired glucose tolerance or elevated fasting blood glucose)” were defined as having diabetes and were included in the subsequent analyses.

### 2.3. Statistical analysis

Categorical data are presented as counts and percentages and compared using chi-square tests, whereas continuous variables are represented as means ± standard deviations and compared using Student *t* tests. Categorical variables with missing data were encoded as “unknown.”^[27]^ Three different models were constructed using logistic regression models, including unadjusted models (model 1), models minimally adjusted for demographic variables (model 2), and fully adjusted models for all covariates listed in Table 1 (model 3). A trend test was performed using quartiles of Mets-IR as continuous variables. Subgroup analyses were performed to assess the consistency of results across different population strata. To evaluate the potential nonlinear association between the incidence of diabetes and Mets-IR, threshold effect analysis and smoothed curve fitting were employed.

**Table 1:** Patient demographics and baseline characteristics.

Characteristics	Diabetes mellitus		*P*
	No, N = 4737	Yes, N = 520	
Age, mean ± SD	61 ± 8	61 ± 7	.833
Gender, n (%)			<.001
Men	2267 (47.9%)	206 (39.6%)	
Women	2470 (52.1%)	314 (60.4%)	
Marital status, n (%)			.883
Married	4125 (87.1%)	454 (87.3%)	
Unmarried	612 (12.9%)	66 (12.7%)	
Residence, n (%)			.088
Rural village	3191 (67.4%)	331 (63.7%)	
Urban community	1546 (32.6%)	189 (36.3%)	
Education, n (%)			.566
Above high school	117 (2.5%)	15 (2.9%)	
High school and below	4620 (97.5%)	505 (97.1%)	
Smoke, n (%)			<.001
No	2801 (59.1%)	350 (67.3%)	
Yes	1936 (40.9%)	170 (32.7%)	
Drink, n (%)			.007
No	2817 (59.5%)	341 (65.6%)	
Yes	1920 (40.5%)	179 (34.4%)	
Cancer, n (%)			.007
No	4710 (99.4%)	511 (98.3%)	
Yes	27 (0.6%)	9 (1.7%)	
Kidney disease, n (%)			<.001
No	4498 (95.0%)	472 (90.8%)	
Yes	239 (5.0%)	48 (9.2%)	
Liver disease, n (%)			.401
No	4579 (96.7%)	499 (96.0%)	
Yes	158 (3.3%)	21 (4.0%)	
Mets-IR, mean ± SD	35 ± 19	40 ± 9	<.001

Mets-IR = metabolic score for insulin resistance, SD = standard deviation.

To assess the robustness of the primary findings, a sensitivity analysis was conducted by additionally excluding participants with cancer, renal disease, hepatic disease, pancreatic disease, and those currently receiving pharmacological treatment for diabetes (including traditional Chinese medicine and Western medicine for diabetes, identified using the corresponding CHARLS variables), resulting in an analytic sample of 4263 individuals. To evaluate the potential impact of unmeasured confounding on the observed associations, an *E*-value analysis was performed. The *E*-value represents the minimum strength of association that an unmeasured confounder would need to have with both the exposure and outcome to fully explain away the observed association.

All statistical analyses were performed using R language (version 4.2.3; R Foundation for Statistical Computing, Vienna, Austria) and EmpowerStats software (X&Y Solutions, Inc., Boston). The level of statistical significance was set at *P* = .05 (two-tailed). This study was carried out in compliance with the Strengthening the Reporting of Observational Studies in Epidemiology reporting guideline.

## 3. Results

### 3.1. Baseline characteristics of study participants

The baseline characteristics of the study participants, stratified by diabetes status, are presented in Table 1. The total sample comprised 5257 individuals, with 520 individuals (9.9%) having diabetes. No significant difference was observed in mean age between the groups (61 ± 8 years for nondiabetic vs 61 ± 7 years for diabetic; *P* = .833). However, significant differences (*P* < .001) were noted for gender, smoking, and kidney disease. Specifically, the diabetic group had a higher proportion of women (60.4% vs 52.1%), a lower proportion of current smokers (32.7% vs 40.9%), and a higher prevalence of kidney disease (9.2% vs 5.0%). Significant differences were also found for drinking status (*P* = .007) and cancer history (*P* = .007), with the diabetic group exhibiting a higher proportion of nondrinkers (65.6% vs 59.5%) and a slightly higher prevalence of cancer (1.7% vs 0.6%). A significant difference (*P* < .001) was also evident in the mean value of Mets-IR (40 ± 9 for diabetic vs 35 ± 19 for nondiabetic). No statistically significant differences were found for marital status (*P* = .883), residence type (*P* = .088), education level (*P* = .566), or liver disease history (*P* = .401).

### 3.2. Association between Mets-IR and diabetes

The results of the logistic regression analysis examining the association between the Mets-IR and diabetes are presented in Table 2. When analyzed as a continuous variable, each unit increase in Mets-IR was associated with a 2% higher odds of diabetes in model 1 (odds ratio [OR], 1.02; 95% confidence interval [CI]: 1.01–1.03; *P* = .001), with the association remaining statistically significant after sequential adjustment for sociodemographic and clinical covariates (model 3: OR, 1.01; 95% CI: 1.00–1.02; *P* = .011).When categorized into quartiles (Q1 as reference), a clear dose–response relationship was evident. Compared with the lowest quartile, individuals in the third (Q3) and fourth (Q4) quartiles had significantly higher odds of diabetes across all models; for Q4, the odds were over fivefold higher (model 3: OR, 5.67; 95% CI: 4.17–7.71; *P* < .001). The *P* value for trend was significant (*P* < .001) in all models, indicating a graded increase in diabetes risk with higher Mets-IR levels.

**Table 2:** Association between Mets-IR and diabetes (logistic regression).

Characteristics	Model 1			Model 2			Model 3		
	OR	95% CI	*P*	OR	95% CI	*P*	OR	95% CI	*P*
Mets-IR	1.017	1.007–1.028	.001	1.016	1.005–1.027	.003	1.014	1.003–1.025	.011
Mets-IR									
Q1	–	–		–	–		–	–	
Q2	1.264	0.882–1.812	.202	1.275	0.889–1.828	.187	1.292	0.900–1.854	.165
Q3	2.509	1.817–3.463	<.001	2.521	1.823–3.487	<.001	2.556	1.844–3.541	<.001
Q4	5.589	4.141–7.544	<.001	5.595	4.133–7.574	<.001	5.668	4.169–7.706	<.001
*P* for trend			<.001			<.001			<.001

Model 1, no covariates were adjusted; model 2, adjusted for age and gender; model 3, adjusted for age, gender, marital status, residence, education, smoking, drinking, cancer, kidney disease, and liver disease.

CI = confidence interval, Mets-IR = metabolic score for insulin resistance, OR = odds ratio, Q = quartile.

### 3.3. Nonlinear relationship and threshold effect between Mets-IR and diabetes

**Figure 2.**
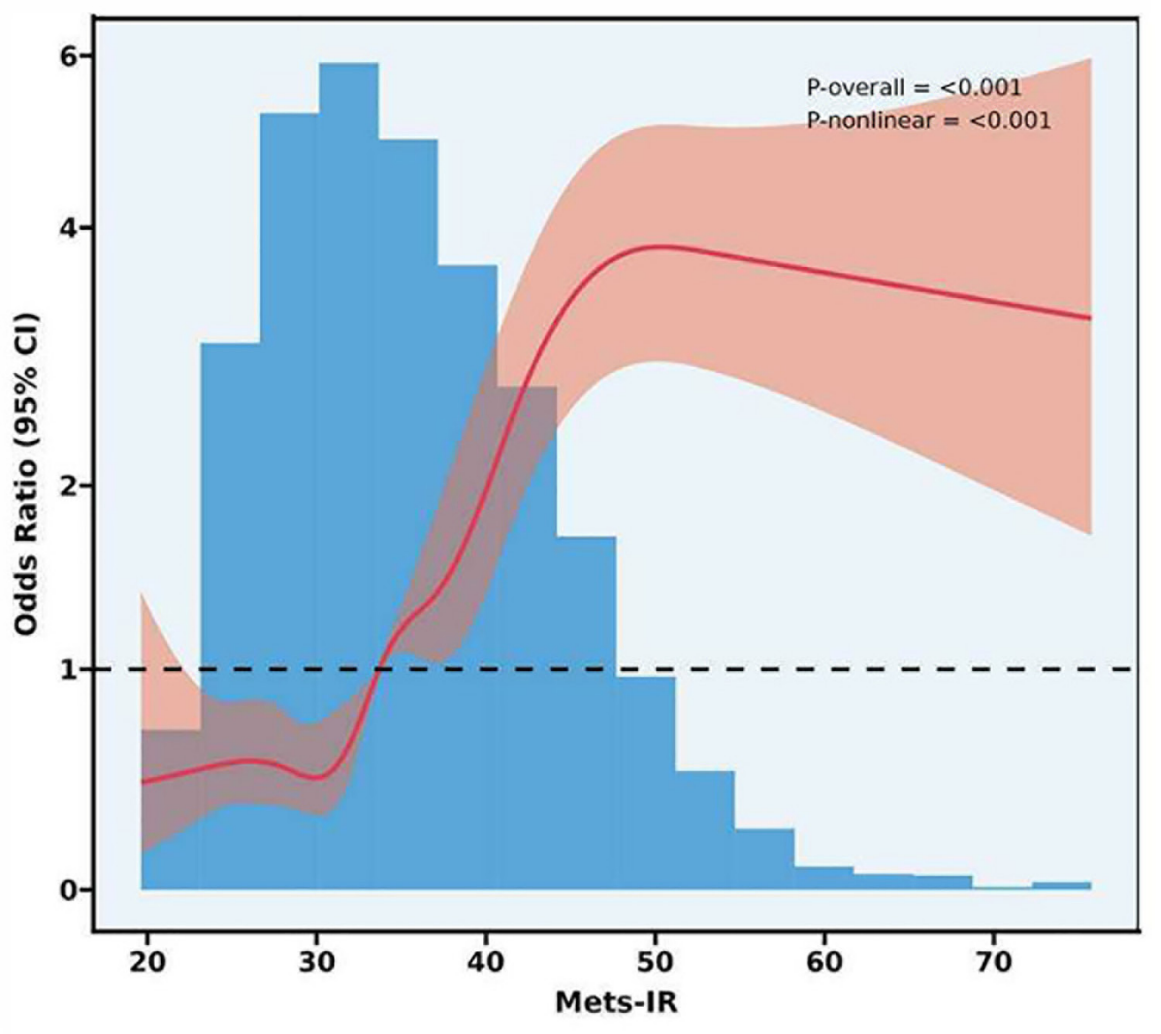
Restricted cubic spline plot between metabolic score for insulin resistance and diabetes.

Threshold effect analysis identified an inflection point at a Mets-IR value of 49.30. Below this threshold, each 1-unit increase in Mets-IR was associated with a 10.6% higher risk of developing DM (OR, 1.11; *P* < .001). Beyond this point, the association was no longer significant (OR, 0.97; *P* = .117). The difference in the slope estimates on either side of the inflection point was statistically significant (log-likelihood ratio test *P* < .001), confirming a nonlinear association (Table 3; Fig. 2).

**Table 3:** Threshold effect analysis of the occurrence of diabetes and Mets-IR

Outcome	New-onset DM	
	OR (95% CI)	*P*
Inflection point	49.30	
<49.30	1.11 (1.09–1.12)	<.001
>49.30	0.97 (0.94–1.01)	.117
Log-likelihood ratio test		<.001

CI = confidence interval, DM = diabetes mellitus, Mets-IR = metabolic score for insulin resistance, OR = odds ratio.

### 3.4. Sensitivity analysis

To further validate the robustness of our findings, a sensitivity analysis was performed after additionally excluding participants with cancer, renal disease, hepatic disease, pancreatic disease, and those currently receiving pharmacological treatment for diabetes (n = 4263). As shown in Table 4, model 1 represents the unadjusted model, and model 2 represents the model adjusted for sociodemographic variables (age and gender). When analyzed as a standardized continuous variable, the OR was 1.26 (95% CI: 1.04–1.54; *P* = .021) in model 1 and 1.23 (95% CI: 1.01–1.50; *P* = .041) in model 2. Quartile analysis consistently demonstrated that individuals in Q4 had the highest odds of diabetes (model 1: OR, 3.80; 95% CI: 2.80–5.15; model 2: OR, 3.73; 95% CI: 2.74–5.08; both *P* < .001), with significant *P* values for trend across both models (*P* < .001), confirming that the dose–response relationship was preserved. Furthermore, the *E*-value for the point estimate was 1.13, indicating that the observed association remained present after accounting for the potential influence of unmeasured confounding factors.

**Table 4:** Sensitivity analysis for the association between Mets-IR and diabetes.

Characteristics	Model 1			Model 2		
	OR	95% CI	*P*	OR	95% CI	*P*
Mets-IR (standardized)	1.26	1.04–1.54	.021	1.23	1.01–1.50	.041
Mets-IR						
Q1	–	–		–	–	
Q2	1.18	0.83–1.69	.360	1.18	0.83–1.70	.358
Q3	1.98	1.43–2.75	<.001	1.98	1.42–2.75	<.001
Q4	3.80	2.80–5.15	<.001	3.73	2.74–5.08	<.001
*P* for trend			<.001			<.001

Model 1, unadjusted; model 2, adjusted for age and gender.

CI = confidence interval, Mets-IR = metabolic score for insulin resistance, OR = odds ratio, Q = quartile,.

### 3.5. Subgroup analyses of the association between Mets-IR and diabetes

**Figure 3.**
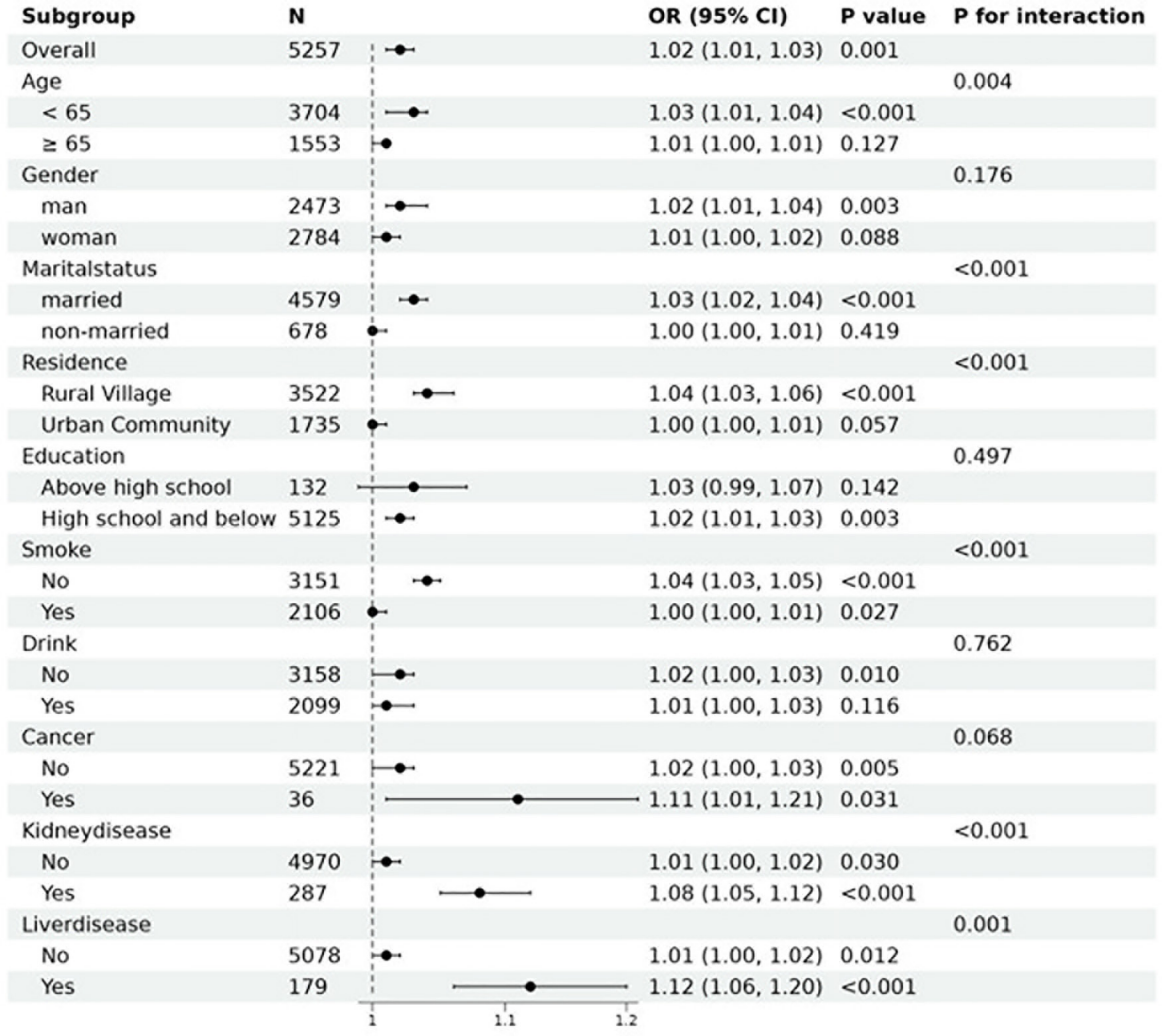
Forest plots among different subgroups.

Subgroup analyses were subsequently performed (Fig. 3). The overall analysis (N = 5257) revealed a statistically significant positive association between the metabolic score for Mets-IR and diabetes, with an OR of 1.02 (95% CI: 1.01–1.03; *P* = .001). Significant interaction effects were observed for age (*P* for interaction = .004), marital status (*P* for interaction < .001), residence (*P* for interaction < .001), smoking status (*P* for interaction < .001), kidney disease (*P* for interaction < .001), and liver disease (*P* for interaction = .001). Specifically, the association was stronger and statistically significant among participants aged <65 years, those who were married, residents of rural villages, nonsmokers, those without kidney disease, and those without liver disease. In contrast, the association was attenuated and nonsignificant among participants aged ≥65 years, the unmarried, urban community residents, and smokers. Notably, among participants with kidney disease (OR, 1.08; 95% CI: 1.05–1.12; *P* < .001) or liver disease (OR, 1.12; 95% CI: 1.06–1.20; *P* < .001), the positive association with Mets-IR was particularly pronounced. No significant interaction effects were identified for gender (*P* for interaction = .176), education level (*P* for interaction = .497), drinking status (*P* for interaction = .762), or cancer history (*P* for interaction = .068).

## 4. Discussion

### 4.1. From obesity to insulin resistance

Obesity is a well-established risk factor for DM,^[28–31]^ with BMI serving as a standard metric for its assessment. Higher BMI is consistently associated with an elevated risk of DM.^[32–34]^ Concurrently, dyslipidemia, a component of metabolic dysregulation, further contributes to this risk.^[35–38]^ A key pathophysiological link between these conditions and diabetes is insulin resistance, where normal or elevated insulin levels fail to elicit an adequate glucose-lowering response, leading to hyperglycemia.^[39–42]^ Consequently, there is a clear clinical need for a simple surrogate measure of insulin resistance, often derived from routine indicators such as blood lipid levels, BMI, and fasting glucose. Mets-IR integrates these components. As individuals with DM typically exhibit higher Mets-IR scores, we hypothesized that a higher Mets-IR could predict new-onset DM.

### 4.2. Nonlinear relationship between Mets-IR and new-onset DM

Research indicates that, after fully adjusting for confounding factors, the risk of new-onset diabetes gradually increases with the increase in Mets-IR. This association remained significant after adjustment for potential confounders, which confirms that Mets-IR can be of great value to the public health system for the prevention and monitoring of DM in the population. We found that the predictive utility of Mets-IR was consistent across subgroups stratified by gender and age. This suggests that Mets-IR is universally predictive of new-onset DM in different genders and different age groups. Through threshold effect analysis and smoothed curve fitting, we identified a nonlinear association between Mets-IR and DM risks. When Mets-IR is <49.30, the likelihood of diabetes is more pronounced with the increase of Mets-IR; when Mets-IR is >49.30, the risk of developing diabetes slows down significantly as Mets-IR increases, but the majority of the population has a Mets-IR of <49.30, which suggests to us that when we have a Mets-IR of <49.30, we should try to minimize the Mets-IR value as much as possible. Given that the majority of the population resides below this threshold, our findings imply that interventions aimed at reducing Mets-IR, primarily via control of its component factors, could be highly effective for population-wide diabetes prevention.

### 4.3. Research contributions and novelty

This study supplements the existing literature in the following aspects. First, it is among the first to prospectively verify the effectiveness of Mets-IR in the middle-aged and elderly population with DM in China. This population has unique metabolic characteristics and risk profiles. Second, it identifies a population-specific threshold effect, offering a potential cutoff for risk stratification that differs from values reported in Mexican or Korean cohorts.^[43–45]^ Finally, our long-term follow-up data elucidate the nonlinear dynamics of Mets-IR in DM progression, providing a novel basis for individualized screening strategies.

### 4.4. Limitations and future directions

There are still several limitations in our study. First, we are based on data from the CHARLS database, from which we screened for people aged ≥50 years. Thus, the findings may not be generalizable to the entire population. Second, many of the indicators were derived from self-reported surveys, which may lead to recall bias. A multicenter intervention trial should be conducted to verify whether reducing Mets-IR can delay the onset of DM. Combining continuous glucose monitoring and multiomics approaches (e.g., exploring links with gut microbiota and inflammatory markers) could elucidate the underlying mechanisms. Developing and externally validating a personalized risk prediction model incorporating Mets-IR across diverse ethnicities is a critical next step.

## 5. Conclusions

This study shows that in people aged ≥50 years, a higher Mets-IR is significantly associated with an increased risk of new-onset DM. This association is nonlinear, with a critical inflection point of 49.30. Below this point, DM risk increases significantly. This pattern is prevalent across different age groups and genders. These findings make Mets-IR a practical and effective tool for identifying individuals at high risk of DM, thereby facilitating early and targeted preventive interventions.

## Author contributions

**Conceptualization:** Baojia Zhang.

**Data curation:** Baojia Zhang.

**Formal analysis:** Baojia Zhang.

**Investigation:** Baojia Zhang.

**Methodology:** Baojia Zhang.

**Project administration:** Baojia Zhang.

**Resources:** Baojia Zhang.

**Software:** Baojia Zhang.

**Validation:** Baojia Zhang.

**Visualization:** Baojia Zhang.

**Writing – original draft:** Baojia Zhang.

**Writing – review & editing:** Baojia Zhang.
